# Robust research tools shed light on the crucial development issue of alcohol harm and enable effective policy adoption

**DOI:** 10.1111/dar.12846

**Published:** 2018-08-13

**Authors:** Natacha Lecours, Greg Hallen

**Affiliations:** ^1^ International Development Research Centre Ottawa Canada

The global epidemic of non‐communicable diseases represents one of the main global health challenges of current times, and a formidable threat to development and economies 1, 2. The harmful use of alcohol is one of the leading risk factors contributing to preventable deaths from non‐communicable diseases; it is a causal factor in 60 types of diseases and injuries and a component cause in 200 others 3. It also negatively affects the well‐being and health of people around those that consume alcohol in a harmful way 4.

As part of Canada's foreign affairs and development efforts, the International Development Research Centre (IDRC) invests in knowledge, innovation and solutions to improve the lives of people in the developing world. The Centre's work has included a focus on non‐communicable disease prevention since 2011, building on 15 years of support to tobacco‐control research. In response to this development challenge, IDRC started to support alcohol research in 2012. Acknowledging the need for evidence from low‐ and middle‐income countries (LMIC) to stimulate policy dialogues and promote action toward international targets, IDRC's funding supported research that addressed gaps in knowledge in the area of population‐wide approaches to prevent or reduce the harmful use of alcohol.

To effectively address the health, safety and socioeconomic problems attributable to alcohol, public health experts and international organisations have recommended a series of actions and policies that countries have a responsibility to formulate, implement, monitor and evaluate. These interventions, along with clear timelines and targets, are well described in the World Health Organization Global Action Plan for the Prevention and Control of Non‐communicable Diseases 2013–2020 5 and the Global strategy to reduce harmful use of alcohol 6. A set of best‐buy non‐communicable disease interventions that are highly cost‐effective and therefore feasible to implement in resource‐constrained settings include regulating alcohol beverage marketing, restricting alcohol availability, reducing demand through taxation and pricing mechanisms and raising awareness of public health problems to ensure support for policies 7. These policies are key to both prevent alcohol‐related harm and a high incidence of non‐communicable diseases and injuries in LMICs 8. Addressing these public health issues is also crucial in any serious effort to move forward the Sustainable Development Goals agenda, including poverty eradication, good health and well‐being, sustainable (and safe) cities and communities and reduced inequality goals, to name just a few 9.

Despite a widespread international consensus on the severity of the burden and on the need for countries to implement cost‐effective solutions, according to the Global Information System on Alcohol and Health, only 39% of World Health Organization Member States report having an alcohol policy at the national level 10. The portfolio of IDRC‐supported projects on alcohol harm reduction highlights some of the most important barriers to policy implementation in LMICs. These include strong industry presence in public debates and participation in policy‐making processes, aggressive marketing and promotion strategies – mostly unregulated and targeting the youth, and influential corporate social responsibility campaigns 11, 12, 13. In addition, a paucity of local evidence on the magnitude of alcohol‐related health and economic burdens, and the potential effectiveness of various measures, act as an additional barrier to intervention prioritisation and policy adoption 14.

The International Alcohol Control (IAC) Study collects and analyses detailed information on these two topics with its two research tools – an extensive survey of the drinking population and a protocol for an analysis of the alcohol policy environment 15, 16. Analyses of these datasets allow countries to improve their understanding of the links between existing consumption patterns and their enablers, a set of implemented policy interventions and subsequent changes in drinking behaviours and consumption patterns. The IAC Study has thus proven extremely helpful in a wide range of contexts across the policy implementation spectrum. For example, in countries where policy progress has been non‐existent or very slow, the IAC tools have allowed research teams to generate either detailed data on consumption (including type, quantity, access and affordability) or on the policy environment (even when it was weak), or both. These results provided an important baseline of local data, which countries have used to initiate public debate and policy dialogue, and on which they will be able to build when policy interventions are considered. Given that the IAC methodology enables both a thorough description of the problems and an assessment of the actual impacts of policy interventions (single or in packages), it has great potential to be an effective aid in policy‐making processes, especially where there is interest and political will.

Through research collaborations and the production of findings which are comparable between countries, the IAC Study has positively contributed to the knowledge base on alcohol‐related harm globally. The cross‐country analyses that the IAC Study has assembled in this Special Issue, in addition to others available elsewhere, directly address the knowledge gaps that create policy barriers in both high‐income countries and LMICs. The analyses show important differences between high‐income country and middle‐income country contexts. For example, one of the studies by Huckle *et al*. 17 found that socioeconomic disadvantage predicted heavy drinking in high‐ but not in middle‐income countries. The authors indicate that this result may relate to the affordability of alcohol, with alcohol being more affordable in high‐income countries even among the most disadvantaged. This in turn points to the importance of affordability measures and analysis. In relation to these, the article from Wall *et al*. 18 provides a useful example of the type of cross‐country price and taxation analyses that can be performed with the IAC tools, and the policy‐relevant findings that they contribute. In this case, the authors observed considerable variation in tax systems and prices across six countries, and despite the complexity of both assessing and implementing alcohol tax systems, they identified important scope to increase rates of taxation to meet international recommendations and have a positive outcome on public health. In addition to socioeconomic variables findings, the article from Chaiyasong *et al*. 19 also underlines the gaps in knowledge that exist with regards to other important variables such as age and gender. These gaps point to a need to further investigate how countries can understand and address specific group vulnerabilities in health and social equity promotion efforts.

As a supporter of research for development, IDRC values the outputs and outcomes of the IAC Study across a wide range of country contexts, and has provided support for the IAC data collection in selected LMICs. IDRC encourages others to invest in opportunities to generate public health, development and economic impacts that can be realised by advancing alcohol control research and policy.
